# Supplementary material for: Multi‐dataset identification of innovative feature genes and molecular mechanisms in keratoconus
Source: J Cell Mol Med. 2024 Sep 19;28(18):e70079. doi: 10.1111/jcmm.70079 (PMC11412914; doi:10.1111/jcmm.70079)
Supplement: Supplementary file 2 — Material S2. [file JCMM-28-e70079-s002.zip › 7.GSEA analysis/2.ATOH7/KEGG_PPAR_SIGNALING_PATHWAY.html]

Details for gene set KEGG\_PPAR\_SIGNALING\_PATHWAY[GSEA]

|  || Dataset | ATOH7.group.cls#h\_versus\_l |
| Phenotype | group.cls#h\_versus\_l |
| Upregulated in class | h |
| GeneSet | KEGG\_PPAR\_SIGNALING\_PATHWAY |
| Enrichment Score (ES) | 0.46607748 |
| Normalized Enrichment Score (NES) | 1.5324796 |
| Nominal p-value | 0.022821577 |
| FDR q-value | 0.28509372 |
| FWER p-Value | 0.733 |
Table: GSEA Results Summary

  

Fig 1: Enrichment plot: KEGG\_PPAR\_SIGNALING\_PATHWAY      
 Profile of the Running ES Score & Positions of GeneSet Members on the Rank Ordered List

  

| SYMBOL | TITLE | RANK IN GENE LIST | RANK METRIC SCORE | RUNNING ES | CORE ENRICHMENT || 1 | MMP1 | na | 439 | 0.567 | 0.0388 | Yes |
| 2 | APOA5 | na | 484 | 0.555 | 0.0878 | Yes |
| 3 | CYP27A1 | na | 577 | 0.525 | 0.1328 | Yes |
| 4 | RXRG | na | 913 | 0.462 | 0.1650 | Yes |
| 5 | FABP7 | na | 1192 | 0.421 | 0.1952 | Yes |
| 6 | PLIN1 | na | 1258 | 0.414 | 0.2309 | Yes |
| 7 | FABP3 | na | 1713 | 0.367 | 0.2511 | Yes |
| 8 | FABP4 | na | 1970 | 0.346 | 0.2752 | Yes |
| 9 | APOA1 | na | 2211 | 0.329 | 0.2981 | Yes |
| 10 | CYP4A22 | na | 2223 | 0.328 | 0.3275 | Yes |
| 11 | ACOX2 | na | 2297 | 0.323 | 0.3547 | Yes |
| 12 | PPARG | na | 2303 | 0.323 | 0.3839 | Yes |
| 13 | OLR1 | na | 2618 | 0.302 | 0.4022 | Yes |
| 14 | ANGPTL4 | na | 2823 | 0.290 | 0.4227 | Yes |
| 15 | PCK2 | na | 2919 | 0.285 | 0.4457 | Yes |
| 16 | GK | na | 3084 | 0.276 | 0.4661 | Yes |
| 17 | PLTP | na | 5885 | 0.174 | 0.4011 | No |
| 18 | CD36 | na | 6081 | 0.169 | 0.4108 | No |
| 19 | PCK1 | na | 6929 | 0.145 | 0.3995 | No |
| 20 | CYP8B1 | na | 7457 | 0.132 | 0.3962 | No |
| 21 | ACSL6 | na | 7613 | 0.128 | 0.4034 | No |
| 22 | ACSL4 | na | 8350 | 0.111 | 0.3922 | No |
| 23 | EHHADH | na | 8475 | 0.109 | 0.3985 | No |
| 24 | ACSL5 | na | 9868 | 0.083 | 0.3659 | No |
| 25 | ACSL3 | na | 10431 | 0.075 | 0.3565 | No |
| 26 | FABP2 | na | 11752 | 0.056 | 0.3235 | No |
| 27 | SLC27A2 | na | 11946 | 0.053 | 0.3227 | No |
| 28 | RXRB | na | 12340 | 0.048 | 0.3157 | No |
| 29 | UBC | na | 12688 | 0.044 | 0.3097 | No |
| 30 | ILK | na | 13116 | 0.039 | 0.3009 | No |
| 31 | ADIPOQ | na | 13485 | 0.035 | 0.2935 | No |
| 32 | ME1 | na | 14451 | 0.026 | 0.2681 | No |
| 33 | APOC3 | na | 14625 | 0.024 | 0.2653 | No |
| 34 | SLC27A1 | na | 14836 | 0.023 | 0.2613 | No |
| 35 | SLC27A6 | na | 15435 | 0.017 | 0.2456 | No |
| 36 | NR1H3 | na | 16313 | 0.010 | 0.2212 | No |
| 37 | SORBS1 | na | 16752 | 0.007 | 0.2092 | No |
| 38 | PPARD | na | 17078 | 0.004 | 0.2002 | No |
| 39 | APOA2 | na | 17638 | -0.001 | 0.1841 | No |
| 40 | DBI | na | 17769 | -0.002 | 0.1806 | No |
| 41 | FABP5 | na | 18330 | -0.007 | 0.1650 | No |
| 42 | LPL | na | 19576 | -0.018 | 0.1307 | No |
| 43 | ACADM | na | 20219 | -0.024 | 0.1144 | No |
| 44 | SLC27A5 | na | 20617 | -0.028 | 0.1054 | No |
| 45 | CPT1C | na | 20712 | -0.029 | 0.1053 | No |
| 46 | SCP2 | na | 21019 | -0.032 | 0.0994 | No |
| 47 | UCP1 | na | 21041 | -0.032 | 0.1017 | No |
| 48 | CYP4A11 | na | 21497 | -0.037 | 0.0919 | No |
| 49 | RXRA | na | 21798 | -0.041 | 0.0870 | No |
| 50 | ACOX3 | na | 21920 | -0.042 | 0.0873 | No |
| 51 | SLC27A4 | na | 22503 | -0.048 | 0.0749 | No |
| 52 | HMGCS2 | na | 23196 | -0.057 | 0.0601 | No |
| 53 | ACSL1 | na | 23435 | -0.061 | 0.0588 | No |
| 54 | CPT1A | na | 23792 | -0.067 | 0.0546 | No |
| 55 | SCD | na | 24053 | -0.070 | 0.0535 | No |
| 56 | PDPK1 | na | 24308 | -0.074 | 0.0529 | No |
| 57 | CPT1B | na | 24648 | -0.080 | 0.0503 | No |
| 58 | PPARA | na | 24700 | -0.081 | 0.0562 | No |
| 59 | ACOX1 | na | 25688 | -0.098 | 0.0366 | No |
| 60 | CPT2 | na | 26293 | -0.111 | 0.0292 | No |
| 61 | ACAA1 | na | 26532 | -0.115 | 0.0328 | No |
| 62 | FABP1 | na | 26875 | -0.122 | 0.0340 | No |
| 63 | SCD5 | na | 27173 | -0.130 | 0.0373 | No |
| 64 | FADS2 | na | 29483 | -0.194 | -0.0118 | No |
| 65 | FABP6 | na | 29594 | -0.197 | 0.0030 | No |
| 66 | CYP7A1 | na | 31395 | -0.263 | -0.0250 | No |
| 67 | AQP7 | na | 33205 | -0.385 | -0.0423 | No |
| 68 | GK2 | na | 33353 | -0.400 | -0.0102 | No |
| 69 | ACADL | na | 34227 | -0.550 | 0.0145 | No |
Table: GSEA details [plain text format]

  

Fig 2: KEGG\_PPAR\_SIGNALING\_PATHWAY      
 Blue-Pink O' Gram in the Space of the Analyzed GeneSet

  

Fig 3: KEGG\_PPAR\_SIGNALING\_PATHWAY: Random ES distribution      
 Gene set null distribution of ES for **KEGG\_PPAR\_SIGNALING\_PATHWAY**
